# Long-term patient reported outcomes following radiation therapy for oropharyngeal cancer: cross-sectional assessment of a prospective symptom survey in patients ≥65 years old

**DOI:** 10.1186/s13014-017-0878-9

**Published:** 2017-09-09

**Authors:** MD Anderson Head and Neck Cancer Symptom Working Group, Salman A. Eraj, Mona K. Jomaa, Crosby D. Rock, Abdallah S. R. Mohamed, Blaine D. Smith, Joshua B. Smith, Theodora Browne, Luke C. Cooksey, Bowman Williams, Brandi Temple, Kathryn E. Preston, Jeremy M. Aymar, Neil D. Gross, Randal S. Weber, Amy C. Hessel, Renata Ferrarotto, Jack Phan, Erich M. Sturgis, Ehab Y. Hanna, Steven J. Frank, William H. Morrison, Ryan P. Goepfert, Stephen Y. Lai, David I. Rosenthal, Tito R. Mendoza, Charles S. Cleeland, Kate A. Hutcheson, Clifton D. Fuller, Adam S. Garden, G. Brandon Gunn

**Affiliations:** 1 Department of Radiation Oncology, Unit 97, The University of Texas MD Anderson Cancer Center, 1515 Holcombe Boulevard, Houston, TX 77030 USA; 2 School of Medicine, The University of Texas Health Science Center at Houston, McGovern School of Medicine, Houston, TX USA; 3 School of Medicine, Texas Tech University Health Sciences Center, Paul L. Foster School of Medicine, El Paso, TX USA; 4 Department of Clinical Oncology and Nuclear Medicine, Faculty of Medicine, University of Alexandria, Alexandria, Egypt; 5 Abilene Christian University, Abilene, TX USA; 6 Department of Head and Neck Surgery, The University of Texas MD Anderson Cancer Center, Houston, TX USA; 7 Department of Medical Oncology, The University of Texas MD Anderson Cancer Center, Houston, TX USA; 8 Department of Epidemiology, Division of OVP, Cancer Prevention and Population Sciences, The University of Texas MD Anderson Cancer Center, Houston, TX USA; 9 Department of Neurosurgery, Division of Surgery, The University of Texas MD Anderson Cancer Center, Houston, TX USA; 10 Department of Symptom Research, Division of Internal Medicine, The University of Texas MD Anderson Cancer Center, Houston, TX USA; 11 Medical Physics Program, The University of Texas Graduate School of Biomedical Sciences, Houston, TX USA

**Keywords:** Oropharynx, Symptoms, Patient reported outcomes

## Abstract

**Background:**

Given the potential for older patients to experience exaggerated toxicity and symptoms, this study was performed to characterize patient reported outcomes in older patients following definitive radiation therapy (RT) for oropharyngeal cancer (OPC).

**Methods:**

Cancer-free head and neck cancer survivors (>6 months since treatment completion) were eligible for participation in a questionnaire-based study. Participants completed the MD Anderson Symptom Inventory-Head and Neck module (MDASI-HN). Those patients ≥65 years old at treatment for OPC with definitive RT were included. Individual and overall symptom severity and clinical variables were analyzed.

**Results:**

Of the 79 participants analyzed, 82% were male, 95% white, 41% T3/4 disease, 39% RT alone, 27% induction chemotherapy, 52% concurrent, and 18% both, and 96% IMRT. Median age at RT was 71 yrs. (range: 65–85); median time from RT to MDASI-HN was 46 mos. (2/3 > 24 mos.). The top 5 MDASI-HN items rated most severe in terms of mean (±SD) ratings (0–10 scale) were dry mouth (3.48 ± 2.95), taste (2.81 ± 3.29), swallowing (2.59 ± 2.96), mucus in mouth/throat (2.04 ± 2.68), and choking (1.30 ± 2.38) reported at moderate-severe levels (≥5) by 35, 29, 29, 18, and 13%, respectively. Thirty-nine % reported none (0) or no more than mild (1–4) symptoms across all 22 MDASI-HN symptoms items, and 38% had at least one item rated as severe (≥7). Hierarchical cluster analysis resulted in 3 patient groups: 1) ~65% with ranging from none to moderate symptom burden, 2) ~35% with moderate-severe ratings for a subset of classically RT-related symptoms (e.g. dry mouth, mucus, swallowing) and 3) 2 pts. with severe ratings of most items.

**Conclusions:**

The overall long-term symptom burden seen in this older OPC cohort treated with modern standard therapy was largely favorable, yet a higher symptom group (~35%) with a distinct pattern of mostly local and classically RT-related symptoms was identified.

**Electronic supplementary material:**

The online version of this article doi: (10.1186/s13014-017-0878-9) contains supplementary material, which is available to authorized users.

## Background

Radiation therapy (RT) (+/− concurrent systemic therapy) is a well-accepted treatment for oropharyngeal carcinoma (OPC). However, the optimal treatment for older patients is not well defined [1]. In a large-scale meta-analysis, the survival benefit of adding chemotherapy to local regional therapy for OPC was shown to diminish with increasing age [2]. The lack of observed benefit in older patients could be due to increased treatment-related toxicity, poorer treatment tolerance, or confounding comorbid conditions, all potentially offsetting any survival benefit. Late sequelae of therapy remain concerning for older populations due to the potential of irreversible functional decline due to limited physical reserves or poor compensatory mechanisms [3]. Additionally, symptoms may be experienced synergistically along with age-related functional decline, thus compounding morbidity from treatment [4].

There is now clinical research emphasis on treatment de-escalation and toxicity reduction strategies in OPC, particularly for those with more favorable/human papillomavirus(HPV)-related disease, as the impact of late toxicities associated with current standard therapies in survivors is now recognized [5]. While HPV-related OPC skews younger, most OPCs in older patients are also HPV-related and incidence in this subset of patients is increasing. With highly curable disease, the importance of long-term toxicity reduction in older patients with OPC should be emphasized [6, 7].

Patients with OPC are increasingly being considered for primary surgical management using transoral and robotic surgery or de-escalation strategies with the goal of maintaining cure rates and reducing toxicity compared to current RT-based approaches [8]. At present, few prospectively collected late toxicity assessment series of OPC survivors treated with modern RT techniques (e.g. intensity modulated radiation therapy(IMRT)) exist to serve as benchmarks for estimation of symptom differentials for patients treated with alternate modalities (e.g. surgery) or advanced RT techniques (e.g. intensity modulated proton therapy).

To help define optimal treatment for older patients in the setting of continually evolving and potentially competing treatment modalities, OPC site-, treatment-, and age-specific patient reported outcomes (PROs) are needed to inform patients, clinicians, and investigators. Thus, we analyzed results of a validated multi-symptom PRO instrument in older OPC survivors in order to characterize the post-therapy symptom experience in patients treated at a tertiary academic medical center.

Consequently, the specific aims of the current study were to:

- Characterize the late patient reported symptom profile of patients ≥65 years old
- Identify potential demographic, disease, treatment-related, and comorbidity-related factors associated with long-term symptom severity
- Explore symptom burden differences by selected patient/treatment subgroups, including by tumor sub-site and receipt of systemic therapy
- Generate testable hypotheses for future clinical research.

## Methods

### Study design

As part of a large-scale Institutional Review Board-approved programmatic prospective symptom survey, adults (≥18 years old) previously treated for head and neck cancer without evidence of active disease and who completed initial therapy more than 6 months previous were eligible for this symptom assessment. Study-specific informed consent was provided by all participants, who then completed the MD Anderson Symptom Inventory-Head and Neck module (MDASI-HN.) Patient demographic, tumor, and treatment characteristics were extracted from medical records, and patient performance status (PS) and comorbidity burden was estimated at the time of treatment according to the ECOG scale and age-adjusted Charlson comorbidity index (CCI), respectively. The CCI assesses 19 comorbid conditions weighted by potential to influence mortality and is used to evaluate the impact of comorbidity on survival and toxicity [9, 10].

Patients targeted for this analysis included those ≥65 years old at the time of definitive RT for OPC. At least 6 months from completion of therapy to MDASI-HN completion was specified in order to allow for stabilization/resolution of acute effects of therapy, allowing focused reporting on more late sequelae [11]. Patients who received RT for recurrence or second primary were excluded. Patients that underwent definitive or salvage surgery at the site of primary disease at any point prior to questionnaire completion were also excluded, excepting patients with a neck dissection (either planned, or as a function of our standardized surveillance protocol) or pre-therapy tonsillectomy [12].

### MDASI-HN module

Patient reported outcomes were detailed by the MDASI-HN, a previously validated, brief, patient-reported outcome assessment tool [13–15]. It contains 28-items consisting of three subscales: 13 core items rating the severity of general symptoms common to all cancers, nine items specific to the MDASI-HN questionnaire, and 6 items concerning how severely symptoms interfere with activities of daily living. The core and head and neck cancer specific items are rated on a 0–10 (“not present” to “as bad as you can imagine”) numeric scale indicating the presence and severity of the symptom. The interference items are rated on a 0–10 numeric scale from “did not interfere” to “interfered completely.”

### Statistical methods

Cumulative symptom burden was characterized by aggregate MDASI-HN symptom score with secondary analysis of specific symptom items. A previously utilized method of patient grouping was used: symptom free (all ratings 0), no more than mild (all ratings <5), no more than moderate (all ratings <7), and severe (at least one item with rating ≥ 7) [16]. Individual item severity was rated using a similar scheme: none (rating 0), mild (rating 1–4), moderate (5–6), and severe (≥7). Grouped and individual MDASI-HN items were tabulated and the proportions of patients reporting each level of symptom severity were presented graphically as heat maps for the entire cohort, as well as for clinical subgroups of interest, hypothesized to have different levels of symptom severity (tumor subsite, T-category, and receipt of concurrent systemic therapy).

The item severity rating means were compared using Wilcoxon rank-sum tests and the proportions of the item severity ratings were compared by Pearson’s chi-squared test, or Fischer’s exact test. Univariate and multivariate regression analysis were performed with the aggregate MDASI-HN symptom items and with composite of the top 5 symptom items as continuous variables against the following variables: sex, race, cancer subsite, receipt of systemic therapy, RT dose, T stage 1–2 vs. 3–4, N stage 0–1 vs. 2+, unilateral radiotherapy, neck dissection, smoking status, and CCI. Variables with a *p*-value <0.3 on univariate analysis were included in multivariate analysis and a non-Bonferonni corrected *p*-value of 0.05 was used as the cutoff for significance, owing to the hypothesis-generating nature of this dataset. Patient clusters were defined by hierarchal cluster analysis and each patient’s individual item ratings are displayed via heat map.

## Results

### Patients

Patient demographic, disease and treatment-related characteristics for the 79 participants are shown in Table 1. Sixty-seven percent completed the MDASI-HN ≥2 years since treatment completion and 89% ≥1 year. Based on the AJCC 7th edition stage grouping, the number of patients falling into stage I, II, III, IVA, and IVB were 9 (11%), 5 (6%), 7 (9%), 57 (72%), and 1 (1%), respectively [17]. Of the 39 tumors tested, 87% were considered HPV-positive, either by p16 or HPV DNA detection. Pretreatment ECOG PS, was available for 68% of the cohort, and 52% were ECOG 0, 39% ECOG 1, and 9% ECOG 2–3.

**Table 1:** Patient demographic, disease, and treatment-related characteristics for the entire study cohort (*n* = 79)

Characteristics	*n* (%)
Male sex	65 (82)
Age at time of RT (years)	
Median	71
Range	65–85
Time from end of RT to MDASI-HN completion (months)	
Median	46
Range	6–117
Race	
White	75 (95)
Black	2 (3)
Hispanic	2 (3)
Patient smoking status	
Former	40 (51)
Current	11 (14)
Never	28 (35)
Tumor subsite	
Base of tongue	45 (57)
Tonsil	32 (41)
Soft palate	1 (1)
Pharyngeal wall	1 (1)
T-category	
T1	22 (28)
T2	25 (32)
T3	21 (27)
T4	11 (14)
N-category	
N0	12 (15)
N1	14 (18)
N2a	9 (11)
N2b	31 (39)
N2c	12 (15)
N3	1 (1)
Age-adjusted Charlson comorbidity index	
Mean ± standard deviation	2.62 ± 0.61
Treatment sequence	
RT alone	31 (39)
CCRT	27 (34)
IC → CCRT	14 (18)
IC → RT	7 (9)
RT dose (Gray)	
Mean ± standard deviation	68.4 ± 2.12

*RT* radiation therapy, *CCRT* concurrent chemoradiation therapy, *IC* induction chemotherapy

Twelve patients (15%) in the cohort underwent a neck dissection as part of their therapy. Of these, 3 were prior to and 9 were after RT. Seventy-six (96%) received IMRT and 11 (14%) received unilateral neck RT for lateralized primaries of the tonsillar fossa. All of the 21 (27%) patients that received induction chemotherapy received combinations of platinum and taxane-based therapies. Of those who received CCRT (concurrent chemoradiotherapy) (41 patients, 52%), the most utilized single agents were cisplatin in 39%, followed by carboplatin in 29%, and cetuximab in 22%.

The comorbidity burden at time of treatment was measured by age-adjusted CCI and had a median of ~3 (IQR 2–4) with 82% of the quantified comorbidity burden coming from age adjustment. The most common non-age-related comorbidity was diabetes mellitus type II in 13 patients (16%), followed by cerebrovascular disease in 7 patients (9%) and chronic pulmonary disease in 6 (8%). Beyond CCI measures, 37 patients (47%) had hypertension and 43 (54%) had general cardiac comorbidity (defined as the presence or history of hypertension, coronary artery disease, myocardial infarction, or congestive heart failure).

### Symptoms reports

**Fig. 1:**
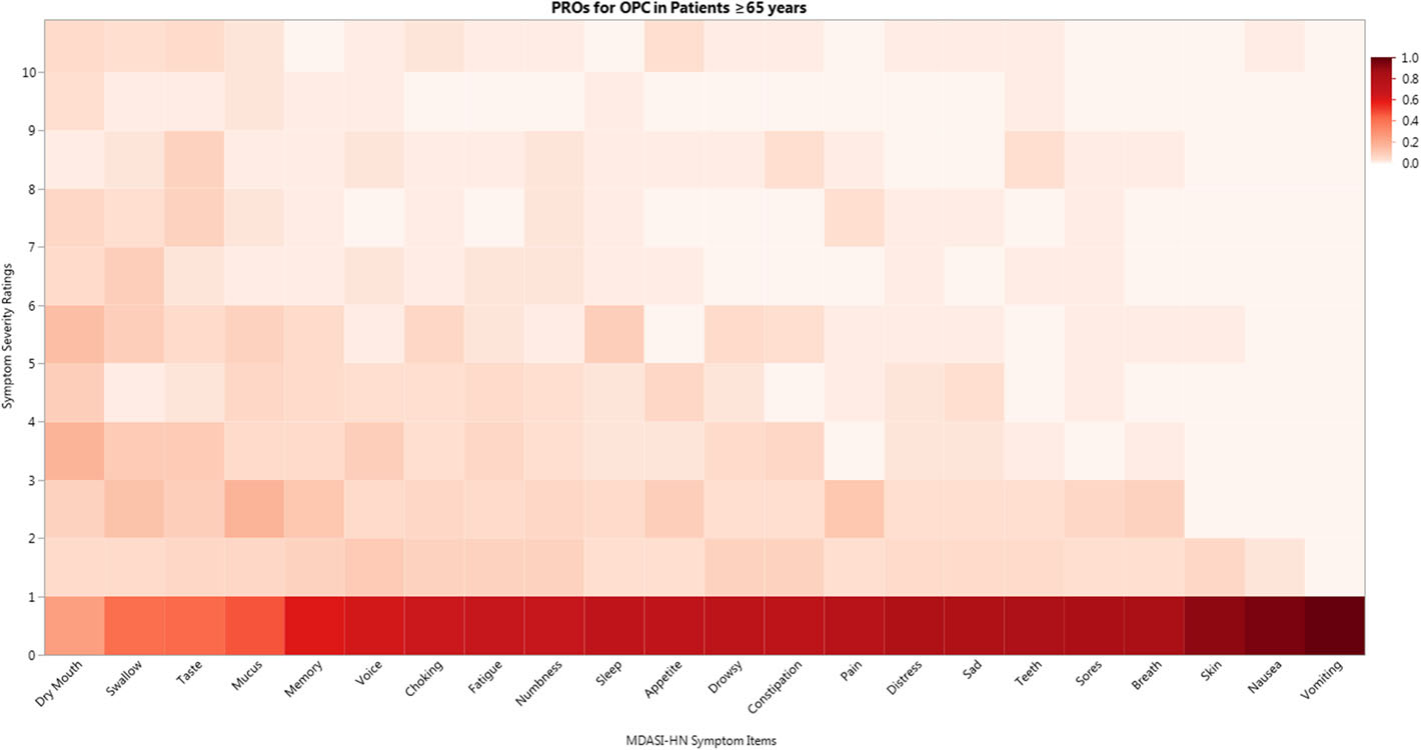
Heat map depicting the proportion of patients experiencing each level of symptom severity (0–10) for the 22 individual MDASI-HN symptom items for the entire study cohort (*n* = 79)

The mean individual, subscale, and composite MDASI-HN item ratings for the entire cohort are shown in Table 2. A heat map of the proportion of the entire cohort experiencing each level of symptom severity for the 22 MDASI-HN symptom items is shown in Fig. 1. Overall, the five most highly rated items by mean ± SD were dry mouth (3.48 ± 2.95), problems tasting food (2.81 ± 3.29), difficulty swallowing/chewing (2.59 ± 2.96), problem with mucus in mouth/throat (2.04 ± 2.68), and choking/coughing (1.30 ± 2.38), reported at moderate-severe levels (≥5) by 35, 29, 29, 18, and 13% of patients, respectively. Of the entire cohort, 9% were symptom free (all 22 symptom items rated zero), 30% had no more than mild symptoms (<5), and 38% had at least one item rating that was severe (≥7).

**Table 2:** Mean individual MDASI-HN symptom item and symptom interference ratings (and standard deviation [SD]) by order of decreasing mean severity for the entire study cohort (*n* = 79)

	Mean	SD
MDASI-HN core items		
Dry mouth	3.48	2.95
Difficulty remembering	1.29	2.09
Numbness/tingling	1.19	2.30
Sleep disturbance	1.14	2.18
Lack of appetite	1.10	2.36
Fatigue	1.08	2.04
Drowsiness	0.89	1.92
Pain	0.75	1.78
Distress	0.66	1.76
Sadness	0.63	1.70
Shortness of breath	0.39	1.20
Nausea	0.15	1.13
Vomiting	0.00	0.00
*Subtotal (13 MDASI-HN core items)*	*0.98*	*1.80*
MDASI-HN-specific items		
Problem tasting food	2.81	3.29
Difficulty swallowing/chewing	2.59	2.96
Problem with mucus in mouth/throat	2.04	2.68
Choking/coughing	1.30	2.38
Difficulty with voice	1.28	2.30
Constipation	0.96	2.14
Problem with teeth/gums	0.78	2.24
Mouth/throat sores	0.54	1.56
Skin pain/burning/rash	0.13	0.61
*Subtotal (9 MDASI-HN-specific items)*	*1.38*	*2.24*
*Total (all 22 symptom items)*	*1.18*	*2.02*
Symptom interference items		
Normal work	0.91	2.03
Enjoyment of life	0.90	2.02
Walking	0.85	2.23
General activity	0.71	1.87
Mood	0.53	1.55
Relations with others	0.49	1.75
*Total (6 symptom interference items)*	*0.73*	*1.91*

For the 30 patients rating at least one item as severe, the median number of items that were rated severe was 2 (IQR 1–4). In this subgroup, the five most highly rated items mean ± SD were dry mouth (5.83 ± 3.00), problems with taste (5.73 ± 3.34), difficulty swallowing/chewing (4.33 ± 3.42), problem with mucus in mouth/throat (3.37 ± 3.41), and problem with voice (2.43 ± 3.18), a result similar to that of the entire cohort. This indicates the presence of low overall item scoring with individual items rated severe driving the mean. Notably, difficulty swallowing/chewing, choking/coughing, and fatigue were reported at severe levels (≥7), in 11%, 5%, and 3%, respectively. Overall, only 2 patients had a feeding tube present at the time of MDASI-HN.

### Clinical subgroups

**Fig. 2:**
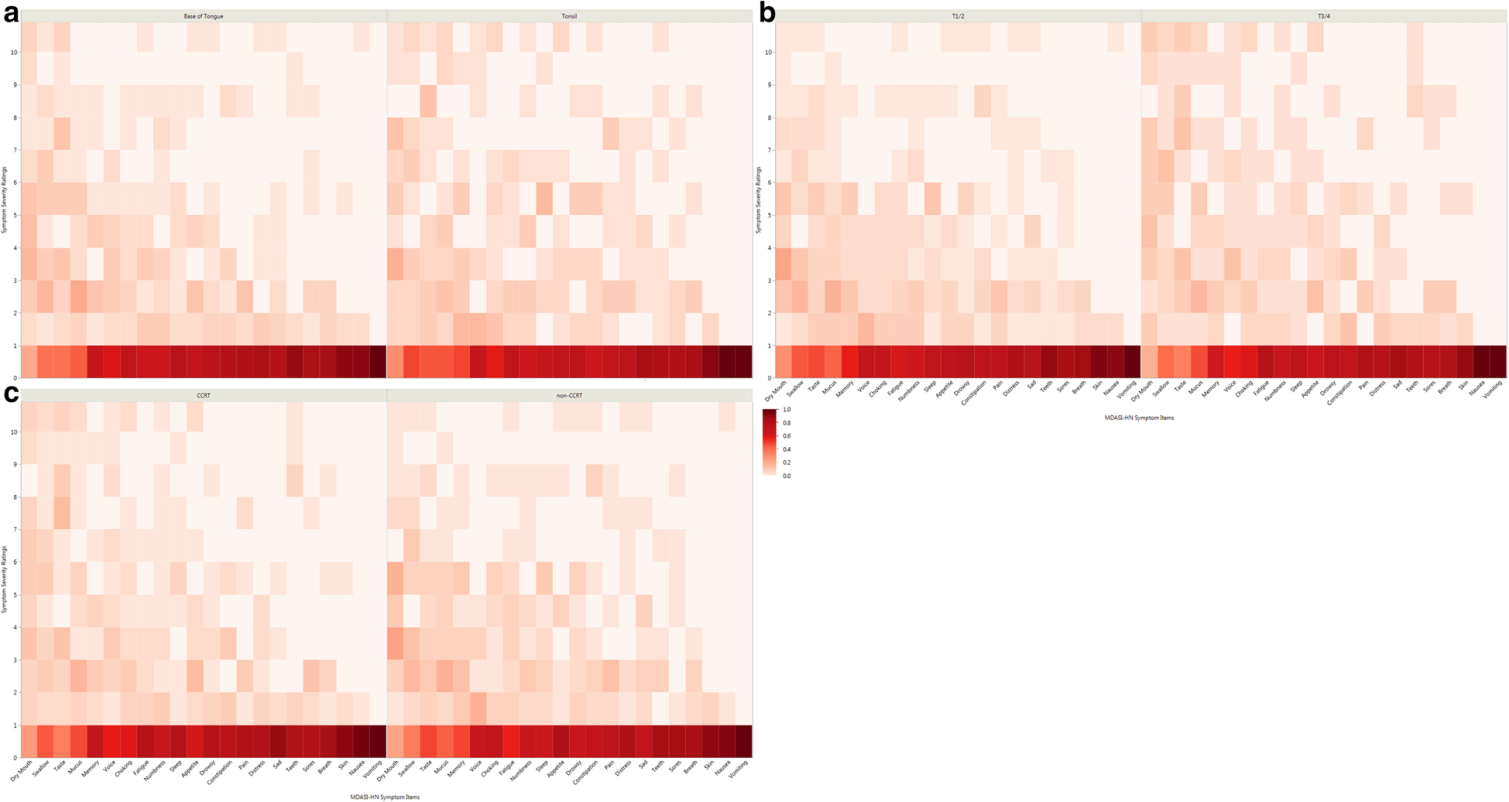
Heat map depicting the proportion of patients experiencing each level of symptom severity (0–10) for the 22 individual MDASI-HN symptom items for those with base of tongue versus tonsil primaries (panel **a**), those who had T1/2 versus T3/4 disease, and those who received CCRT versus those who did not (panel **c**). Figure legend adjacent to panel **c** applies to panels **a**-**c**

The proportion of patients experiencing each level of symptom severity for the 22 individual MDASI-HN symptom items comparing clinical subgroups of interest (primary site, T-category, and receipt of concurrent chemotherapy) are shown as heat maps in Fig. 2 and the proportions reporting these at moderate-severe levels are compared in Additional file 1: Table S1. Likewise, the proportions reporting severe levels are compared in Additional file 2: Table S2. Statistically significant differences in the proportions of patients reporting moderate-severe levels were identified for a limited number of symptoms in subgroup comparisons (difficulty with voice and problems with teeth/gums were worse for T3/4 and distress, problem tasting food, and difficulty with voice were worse for those receiving concurrent chemotherapy), and no differences were noted in tumor subsite comparisons. Statistically significant differences in the proportions of patients reporting severe levels were also identified for a limited number of symptoms in subgroup comparisons (problem tasting food, difficulty with voice, problem with teeth/gums were worse for T3/4 and problem tasting food and constipation were worse for those receiving concurrent chemotherapy), and again no differences were noted in by tumor subsite comparisons. To explore symptom differentials by age, we compared the proportions of patients reporting moderate-severe level symptoms (≥5), comparing those ≥75 versus <75 years old, and using this cut-point, there were no statistically significant differences detected.

**Fig. 3:**
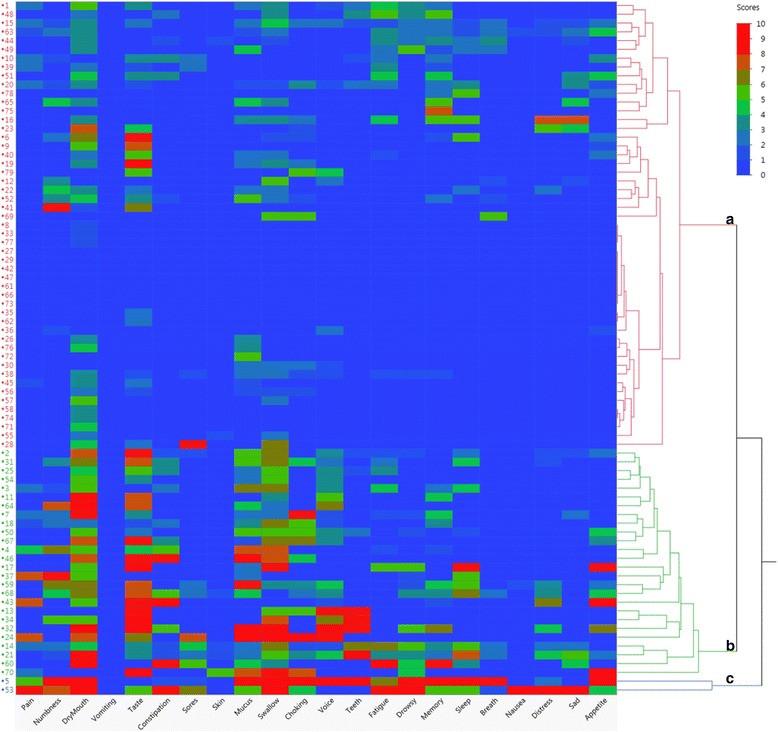
Heat map of the severity ratings for the 22 individual MDASI-HN symptom items, for each individual patient (numbered 1–79), and grouped by hierarchal cluster analysis of patients with accompanying dendrogram (far right of the figure). Three patient clusters were identified (labelled A, B, and C). The dendrogram shows how similarly individual patients reported symptom severity across all 22 items. The lines that join further to the left side of the dendrogram suggest these patients reported similar symptom pattern and severity ratings

Hierarchical cluster analysis results are presented in Fig. 3. Cluster A comprised the majority (64%), with a subset symptom free and the majority with no more than moderate ratings for a limited number of items. The distribution of these items fell into two sub-clusters, one centered around more general, constitutional symptoms of fatigue, memory, drowsiness, and sadness, and another centered around more classical RT-related toxicities, such as choking/coughing, dry mouth, problem with mucus in mouth/throat, difficulty swallowing/chewing, and problem tasting food. Cluster B (33%) patients had a more moderate-severe symptom burden with a heterogeneous distribution of several severely rated items. There again was a sub-cluster centered around moderate global symptoms, yet more broadly spread than cluster A. Similar to cluster A were distinct bands, yet more severely rated, for the same classic RT-related toxicities observed in cluster A. Cluster C formed the small minority (~2%), with essentially severe ratings for the majority of all 22 items.

### Clinical correlates of symptom severity

On univariate analysis, none of the interrogated variables were shown to be associated with increased composite symptom scores at the *p* < 0.05 level (all 22 items). On univariate analysis with a composite score of the top 5 symptoms, RT dose (*p* < 0.03) and T-category (*p* < 0.04) were significantly associated, but on multivariate analysis, neither of these were found to retain statistical significance. Full results of univariate and multivariate analysis are provided in Additional file 3: Table S3.

## Discussion

This prospective survivorship assessment study of older patients treated with (chemo)RT for OPC using contemporary standard approaches demonstrates a symptom profile which is generally favorably with identifiable subsets of patients with distinct post-therapy symptom constellations. While no one item or items had moderate-severe intensity recorded by a majority of respondents, items for dry mouth, difficulty swallowing/chewing, and taste received notable pluralities, with 35%, 29% and 29% of survivors reporting moderate-severe levels, respectively (Additional file 1: Table S1). Overall, these highest intensity symptom items reported were consistent with “classic” RT-related normal tissue late toxicities, namely xerostomia and dysphagia.

The majority of respondents were free from moderate-severe xerostomia, attributable to the well-characterized, more conformal, treatment offered by IMRT, yet even so, a detectable minority of 16% of the cohort reported severe ratings for dry mouth [18]. Likewise, approximately 10% of survivors reported severe late problems with dysphagia-related symptoms. The correspondingly lower proportion of patients with PEG tube at time of MDASI-HN suggest that the use of objective clinical endpoints alone do not capture the nuanced toxicity burden of this cohort. Additionally, previous studies have shown that even mild late dysphagia, which was present in our cohort, is strongly correlated with overall health related quality of life (HRQOL) (24). Thus, further efforts to improve xerostomia and swallowing dysfunction are likely to yield impactful gain and should be pursued aggressively [19].

Notably, in this older patient cohort, T-category and chemotherapy (either induction and/or concurrent) did not predict strikingly different toxicity profiles. However, individual item-level differences were associated with concurrent chemotherapy and T-category. Specifically, problem tasting food and voice symptoms were more severe in the subgroup of patients with T3-T4 disease which is in line with locally advanced disease infiltrating a larger area of normal structures and resultant larger RT volumes. Cancer subsites (i.e. base of tongue versus tonsil) did not differentially impact the mean item ratings or their distributions, suggesting that this factor alone does not determine the late toxicities experienced by older OPC patients.

Receipt of CCRT was not found to have an association with PROs in univariate analysis. The survival benefit of CCRT versus RT alone in older patients is not generally supported but recent studies have shown that CCRT should not be denied solely based on age [20]. While further study is necessary, the survival benefit and equivalent late toxicity suggested by our findings points towards expansion of CCRT into more aged populations. In a study using SEER-Medicare data regarding OPC in the elderly, as age increased from 70 to 81, treatment shifted toward surgery alone or no treatment [21]. Given the low symptom burden observed within our cohort, it seems subsets of older patients should be considered for standard therapies, as it appears that comorbidity and age did not have a detectable effect upon reported late symptom frequency nor severity.

Hierarchical cluster analysis was also informative with consistent representation of xerostomia at some level in the far majority of patients. While 64% of the cohort presented with generally no to moderate symptom burden, a dismaying ~33% of the cohort still reported moderate-severe ratings for two subsets of symptoms: one centered around classically RT-related symptoms (e.g. dry mouth, problems with mucus, swallowing/chewing, and taste) at moderate-severe ratings, and another around general symptoms (e.g. fatigue, memory, drowsiness) at more moderate ratings. This visualization technique reinforces the continued presence of these two categories of late toxicities and affirms that they tend to co-occur in symptomatic patients, rather than homogenously among the entire cohort.

One of these possible determinants was age-adjusted comorbidity status pre-RT, which has already been shown to have a negative association with overall survival (OS) [22]. Our analysis showed that cohort comorbidity, at least as measured by the age-adjusted CCI in our specific cohort, did not have an association with PROs. This finding may be due to the healthy, homogenous group of older patients that made up our cohort, evidenced by the median age-adjusted CCI of 3, which corresponds to older patients with few comorbidities, and low comorbidity burden outside of age-adjustment [23]. Our study shows that for these older patients judged fit enough to receive curative therapy, PROs remained favorable to a median follow-up period of nearly 4 years (46 months), suggesting that these patients, broadly, maintain global functionality after completion of therapy, as revealed in the low mean symptom interference reports observed here.

Inherent limitations of this analysis include data collected from a large-scale, tertiary, academic cancer institution, acknowledging that, patient self-selection and the patient profile of our specific patient population may not mirror the general OPC population. The patients in our study had comparatively high performance status, low comorbidity burden, and most received standard IMRT. Additionally, survey timing varied widely, 6–117 months, and while previous work has shown that most patients’ toxicity has stabilized 6 months after therapy, it is possible that the varying follow-up periods may not precisely capture late toxicity in this cohort and misrepresent true late toxicity [24]. However, given the low symptom burden observed overall, it is reasonable to presume that toxicity could only have been better with a narrower and later survey acquisition time range. Treatment strategies and expected toxicities differ between AJCC stages and the inhomogeneity of the cohort in this respect is a limitation for external validity. Due to the unbalanced distribution of AJCC stages within our cohort, T1/2 versus T3/4 was use for comparisons as a proxy for locally confined versus locally advanced disease. Baseline symptom data was unavailable, so it is difficult to ascertain what proportion of the symptoms reported were present pre-therapy and potentially persistent versus those that were in fact an actionable secondary side-effect from RT. The standard caveats from any cross-sectional analysis apply in that longitudinal follow-up or the toxicity deltas over time are lacking. Therefore, we recommend the pursuit of studies examining the specific dosimetric effects on normal structures, incorporating tumor HPV status, as well as, analyzing symptom item scores longitudinally with pretreatment baselines, which are already underway at our institution.

Nonetheless our data represents, a large single-site prospective cross-sectional interrogation of late survivorship in older OPC patients who received definitive radiotherapy. It provides a characterized multi-symptom profile for these older patients treated with contemporary techniques, using a standardized approach [25, 26]. These data provide a reference/benchmark dataset against which approaches leveraging alternate modalities (e.g. advanced surgical techniques), or advanced radiation therapy techniques such as proton therapy, may be compared.

## Conclusions

In conclusion, older OPC patients with a median of nearly 4 years from completion of therapy exhibited a broad freedom from global symptoms, with a majority showing no more than mild-to-moderate intensity for experienced symptoms. However, 38% reported at least one severe symptom item. Moderate-severe xerostomia, difficulty tasting food, and dysphagia related symptoms were experienced by a plurality of patients (approximately 1/3 each), but efforts should be made to increase the currently small fraction (9%) of patients who are symptom free survivors.

## Additional files

Additional file 1: Table S1. Proportions of patients reporting moderate to severe (≥5) rating for the 22 MDASI-HN symptom items by clinical subgroups of interest. (DOCX 20 kb)

Additional file 2: Table S2. Proportions of patients reporting severe (≥7) rating for the 22 MDASI-HN symptom items by clinical subgroups of interest. (DOCX 17 kb)

Additional file 3: Table S3. Results of univariate and multivariate analysis comparing MDASI-HN symptom item composite and Top 5 MDASI-HN items by mean composite with clinical variables of interest. (DOCX 16 kb)

## Abbreviations

CCI: Charlson comorbidity index
CCRT: Concurrent chemoradiotherapy
ECOG: Eastern Cooperative Oncology Group
HPV: Human papillomavirus
IMRT: Intensity modulated radiation therapy
MDASI-HN: MD Anderson Symptom Inventory-Head and Neck module
OPC: Oropharyngeal cancer
PRO: Patient reported outcome
PS: Performance status
RT: Radiation therapy
