# Correction to: Long-term patient reported outcomes following radiation therapy for oropharyngeal cancer: cross-sectional assessment of a prospective symptom survey in patients ≥65 years old

**DOI:** 10.1186/s13014-017-0921-x

**Published:** 2017-11-23

**Authors:** MD Anderson Head and Neck Cancer Symptom Working Group, Salman A. Eraj, Mona K. Jomaa, Crosby D. Rock, Abdallah S. R. Mohamed, Blaine D. Smith, Joshua B. Smith, Theodora Browne, Luke C. Cooksey, Bowman Williams, Brandi Temple, Kathryn E. Preston, Jeremy M. Aymard, Neil D. Gross, Randal S. Weber, Amy C. Hessel, Renata Ferrarotto, Jack Phan, Erich M. Sturgis, Ehab Y. Hanna, Steven J. Frank, William H. Morrison, Ryan P. Goepfert, Stephen Y. Lai, David I. Rosenthal, Tito R. Mendoza, Charles S. Cleeland, Kate A. Hutcheson, Clifton D. Fuller, Adam S. Garden, G. Brandon Gunn

**Affiliations:** 1 Department of Radiation Oncology, Unit 97, The University of Texas MD Anderson Cancer Center, 1515 Holcombe Boulevard, Houston, TX 77030 USA; 2 School of Medicine, The University of Texas Health Science Center at Houston, McGovern School of Medicine, Houston, TX USA; 3 School of Medicine, Texas Tech University Health Sciences Center, Paul L. Foster School of Medicine, El Paso, TX USA; 4 Department of Clinical Oncology and Nuclear Medicine, Faculty of Medicine, University of Alexandria, Alexandria, Egypt; 5 Abilene Christian University, Abilene, TX USA; 6 Department of Head and Neck Surgery, The University of Texas MD Anderson Cancer Center, Houston, TX USA; 7 Department of Medical Oncology, The University of Texas MD Anderson Cancer Center, Houston, TX USA; 8 Department of Epidemiology, Division of OVP, Cancer Prevention and Population Sciences, The University of Texas MD Anderson Cancer Center, Houston, TX USA; 9 Department of Neurosurgery, Division of Surgery, The University of Texas MD Anderson Cancer Center, Houston, TX USA; 10 Department of Symptom Research, Division of Internal Medicine, The University of Texas MD Anderson Cancer Center, Houston, TX USA; 11 Medical Physics Program, The University of Texas Graduate School of Biomedical Sciences, Houston, TX USA

## Correction

In the original publication [1] the name of author Jeremy M. Aymard was spelled wrong. The original article was updated to rectify this error.

Incorrect version:

Jeremy M. Aymar.

Correct version:

Jeremy M. Aymard.
