# Supplementary material for: Long-term patient reported outcomes following radiation therapy for oropharyngeal cancer: cross-sectional assessment of a prospective symptom survey in patients ≥65 years old
Source: Radiat Oncol. 2017 Sep 9;12:150. doi: 10.1186/s13014-017-0878-9 (PMC5591495; doi:10.1186/s13014-017-0878-9)
Supplement: Supplementary file 1 — Proportions of patients reporting moderate to severe (≥5) rating for the 22 MDASI-HN symptom items by clinical subgroups of interest. (DOCX 20 kb) [file 13014_2017_878_MOESM1_ESM.docx]

|  | Entire cohort (n=79) | Tumor subsite | | | T-category | | | Receipt of concurrent chemotherapy | | |
| --- | --- | --- | --- | --- | --- | --- | --- | --- | --- | --- |
|  |  | BOT (n=45) | Tonsil (n=32) | p-value | T1/2 (n=47) | T3/4 (n=32) | p-value | CCRT (n=41) | non-CCRT (n=38) | p-value |
|  | %Mod-severe | %Mod-severe | %Mod-severe |  | %Mod-severe | %Mod-severe |  | %Mod-severe | %Mod-severe |  |
| **MDASI-HN core items** | | | | | | | | | | |
| Dry mouth | 35.44% | 35.56% | 34.38% | 0.9148 | 29.79% | 43.75% | 0.2028 | 39.02% | 31.58% | 0.4894 |
| Difficulty remembering | 10.13% | 6.67% | 15.63% | 0.2654 | 10.64% | 9.38% | 1.000 | 4.88% | 15.79% | 0.1449 |
| Numbness/tingling | 10.13% | 11.11% | 9.38% | 1 | 8.51% | 12.50% | 0.7084 | 12.20% | 7.89% | 0.7135 |
| Sleep disturbance | 13.92% | 8.89% | 21.88% | 0.1846 | 12.77% | 15.63% | 0.7186 | 14.63% | 13.16% | 0.8498 |
| Lack of appetite | 6.33% | 4.44% | 9.38% | 0.6438 | 4.26% | 9.38% | 0.3899 | 7.32% | 5.26% | 1 |
| Fatigue | 7.59% | 6.67% | 9.38% | 0.6883 | 10.64% | 3.13% | 0.3924 | 2.44% | 13.16% | 0.1001 |
| Drowsiness | 7.59% | 4.44% | 12.50% | 0.2265 | 8.51% | 6.25% | 1.0000 | 4.88% | 10.53% | 0.4204 |
| Pain | 6.33% | 2.22% | 12.50% | 0.1539 | 4.26% | 9.38% | 0.3899 | 7.32% | 5.26% | 1 |
| Distress | 5.06% | 2.22% | 9.38% | 0.3016 | 8.51% | 0.00% | 0.1426 | 0.00% | 10.53% | 0.0491 |
| Sadness | 3.80% | 4.44% | 3.13% | 1 | 4.26% | 3.13% | 1.0000 | 2.44% | 5.26% | 0.606 |
| Shortness of breath | 2.53% | 0.00% | 6.25% | 0.1695 | 0.00% | 6.25% | 0.1610 | 4.88% | 0.00% | 0.4943 |
| Nausea | 1.27% | 2.22% | 0.00% | 1 | 2.13% | 0.00% | 1.0000 | 0.00% | 2.63% | 0.481 |
| Vomiting | 0.00% | 0.00% | 0.00% | NA | 0.00% | 0.00% | NA | 0.00% | 0.00% | NA |
| **MDASI-HN-specific items** | | | | | | | | | | |
| Problem tasting food | 29.11% | 33.33% | 21.88% | 0.2727 | 21.28% | 40.63% | 0.0631 | 39.02% | 18.42% | 0.044 |
| Difficulty swallowing/chewing | 29.11% | 26.67% | 28.13% | 0.8874 | 21.28% | 40.63% | 0.0631 | 29.27% | 28.95% | 0.975 |
| Problem with mucus in mouth/throat | 17.72% | 15.56% | 18.75% | 0.7123 | 14.89% | 21.88% | 0.4250 | 19.51% | 15.79% | 0.6651 |
| Choking/coughing | 12.66% | 6.67% | 15.63% | 0.2654 | 8.51% | 18.75% | 0.3011 | 14.63% | 10.53% | 0.7389 |
| Difficulty with voice | 8.86% | 6.67% | 9.38% | 0.6883 | 0.00% | 21.88% | 0.0012 | 17.07% | 0.00% | 0.0121 |
| Constipation | 8.86% | 4.44% | 15.63% | 0.1201 | 10.64% | 6.25% | 0.6948 | 4.88% | 13.16% | 0.2521 |
| Problem with teeth/gums | 7.59% | 4.44% | 9.38% | 0.6438 | 2.13% | 15.63% | 0.0373 | 12.20% | 2.63% | 0.2026 |
| Mouth/throat sores | 5.06% | 6.67% | 3.13% | 0.6371 | 4.26% | 6.25% | 1.0000 | 2.44% | 7.89% | 0.3467 |
| Skin pain/burning/rash | 1.27% | 2.22% | 0.00% | 1 | 0.00% | 3.13% | 0.4051 | 2.44% | 0.00% | 1 |
