# Supplementary material for: Long-term patient reported outcomes following radiation therapy for oropharyngeal cancer: cross-sectional assessment of a prospective symptom survey in patients ≥65 years old
Source: Radiat Oncol. 2017 Sep 9;12:150. doi: 10.1186/s13014-017-0878-9 (PMC5591495; doi:10.1186/s13014-017-0878-9)
Supplement: Supplementary file 2 — Proportions of patients reporting severe (≥7) rating for the 22 MDASI-HN symptom items by clinical subgroups of interest. (DOCX 17 kb) [file 13014_2017_878_MOESM2_ESM.docx]

|  | Entire cohort (n=79) | Tumor subsite | | | T-category | | | Receipt of concurrent chemotherapy | | |
| --- | --- | --- | --- | --- | --- | --- | --- | --- | --- | --- |
|  |  | BOT (n=45) | Tonsil (n=32) | p-value | T1/2 (n=47) | T3/4 (n=32) | p-value | CCRT (n=41) | non-CCRT (n=38) | p-value |
|  | %Severe | %Severe | %Severe |  | %Severe | %Severe |  | %Severe | %Severe |  |
| **MDASI-HN core items** | | | | | | | | | | |
| Dry mouth | 16.46% | 15.56% | 18.75% | 0.7123 | 10.64% | 25.00% | 0.091 | 19.51% | 13.16% | 0.4466 |
| Difficulty remembering | 3.80% | 4.44% | 3.13% | 1 | 2.13% | 6.25% | 0.5626 | 2.44% | 5.26% | 0.606 |
| Numbness/tingling | 6.33% | 6.67% | 6.25% | 1 | 4.26% | 9.38% | 0.3899 | 7.32% | 5.26% | 1 |
| Sleep disturbance | 3.80% | 4.44% | 3.13% | 1 | 2.13% | 6.25% | 0.5626 | 4.88% | 2.63% | 1 |
| Lack of appetite | 5.06% | 4.44% | 6.25% | 1 | 4.26% | 6.25% | 1 | 4.88% | 5.26% | 1 |
| Fatigue | 2.53% | 4.44% | 0.00% | 0.5079 | 4.26% | 0.00% | 0.5118 | 0.00% | 5.26% | 0.2282 |
| Drowsiness | 2.53% | 2.22% | 3.13% | 1 | 2.13% | 3.13% | 1 | 2.44% | 2.63% | 1 |
| Pain | 5.06% | 2.22% | 9.38% | 0.3016 | 4.26% | 6.25% | 1 | 4.88% | 5.26% | 1 |
| Distress | 2.53% | 2.22% | 3.13% | 1 | 4.26% | 0.00% | 0.5118 | 0.00% | 5.26% | 0.2282 |
| Sadness | 2.53% | 2.22% | 3.13% | 1 | 4.26% | 0.00% | 0.5118 | 0.00% | 5.26% | 0.2282 |
| Shortness of breath | 1.27% | 0.00% | 3.13% | 0.4156 | 0.00% | 3.13% | 0.4051 | 2.44% | 0.00% | 1 |
| Nausea | 1.27% | 2.22% | 0.00% | 1 | 2.13% | 0.00% | 1 | 0.00% | 2.63% | 0.481 |
| Vomiting | 0.00% | 0.00% | 0.00% | NA | 0.00% | 0.00% | NA | 0.00% | 0.00% | NA |
| **MDASI-HN-specific items** | | | | | | | | | | |
| Problem tasting food | 21.52% | 22.22% | 18.75% | 0.7113 | 10.64% | 37.50% | 0.0043 | 34.15% | 7.89% | 0.0058 |
| Difficulty swallowing/chewing | 11.39% | 8.89% | 15.63% | 0.4772 | 8.51% | 15.63% | 0.4732 | 12.20% | 10.53% | 1 |
| Problem with mucus in mouth/throat | 8.86% | 4.44% | 15.63% | 0.1201 | 6.38% | 12.50% | 0.4323 | 12.20% | 5.26% | 0.4338 |
| Choking/coughing | 5.06% | 4.44% | 6.25% | 1 | 2.13% | 9.38% | 0.2978 | 7.32% | 2.63% | 0.6163 |
| Difficulty with voice | 5.06% | 0.00% | 9.38% | 0.0678 | 0.00% | 12.50% | 0.0239 | 9.76% | 0.00% | 0.1165 |
| Constipation | 5.06% | 4.44% | 6.25% | 1 | 8.51% | 0.00% | 0.1426 | 0.00% | 10.53% | 0.0491 |
| Problem with teeth/gums | 6.33% | 4.44% | 6.25% | 1 | 0.00% | 15.63% | 0.0089 | 12.20% | 0.00% | 0.0555 |
| Mouth/throat sores | 2.53% | 2.22% | 3.13% | 1 | 0.00% | 6.25% | 0.161 | 2.44% | 2.63% | 1 |
| Skin pain/burning/rash | 0.00% | 0.00% | 0.00% | NA | 0.00% | 0.00% | NA | 0.00% | 0.00% | NA |
