# Supplementary material for: Long-term patient reported outcomes following radiation therapy for oropharyngeal cancer: cross-sectional assessment of a prospective symptom survey in patients ≥65 years old
Source: Radiat Oncol. 2017 Sep 9;12:150. doi: 10.1186/s13014-017-0878-9 (PMC5591495; doi:10.1186/s13014-017-0878-9)
Supplement: Supplementary file 3 — Results of univariate and multivariate analysis comparing MDASI-HN symptom item composite and Top 5 MDASI-HN items by mean composite with clinical variables of interest. (DOCX 16 kb) [file 13014_2017_878_MOESM3_ESM.docx]

| **Univariate & Multivariate Analysis** | | | |
| --- | --- | --- | --- |
| **MDASI-HN symptom item composite** | **Variables tested** | **p-value on univariate** | **p-value on multivariate** |
|  | Sex (M/F) | 0.8407 |  |
|  | Race (white Y/N) | 0.2584 | 0.2122 |
|  | Cancer subsite(BOT vs. tonsil) | 0.5179 |  |
|  | Receipt of chemotherapy (Y/N) | 0.9936 |  |
|  | Total RT dose (continuous) | 0.2529 | 0.5784 |
|  | T-category (T1/2 vs. 3/4) | 0.2016 | 0.6399 |
|  | N-category (N0-1 vs. N2+) | 0.9030 |  |
|  | Receipt of unilateral radiation (Y/N) | 0.5132 |  |
|  | neck dissection (Y/N) | 0.7088 |  |
|  | Smoking history (Y/N) | 0.4535 |  |
|  | Age-adjusted CCI | 0.2570 | 0.3159 |
| **Top 5 MDASI-HN items by mean composite** | **Variables tested** | **p-value on univariate** | **p-value on multivariate** |
|  | Sex (M/F) | 0.9089 |  |
|  | Race (white Y/N) | 0.1666 | 0.1599 |
|  | Cancer subsite(BOT vs. tonsil) | 0.9123 |  |
|  | Receipt of chemotherapy (Y/N) | 0.4278 |  |
|  | Total RT dose (continuous) | 0.0228 | 0.3423 |
|  | T-category (T1/2 vs. 3/4) | 0.0334 | 0.3447 |
|  | N-category (N0-1 vs. N2+) | 0.2293 | 0.4715 |
|  | Receipt of unilateral radiation (Y/N) | 0.0701 | 0.5136 |
|  | neck dissection (Y/N) | 0.4365 |  |
|  | Smoking history (Y/N) | 0.5356 |  |
|  | Age-adjusted CCI | 0.783 |  |
